# Chemotherapy‐Sparing Strategies in Follicular Lymphoma: Emerging Targeted and Immune‐Based Approaches

**DOI:** 10.1111/ejh.70105

**Published:** 2025-12-26

**Authors:** Enrica Antonia Martino, Santino Caserta, Mamdouh Skafi, Maria Eugenia Alvaro, Antonella Bruzzese, Nicola Amodio, Eugenio Lucia, Virginia Olivito, Caterina Labanca, Francesco Mendicino, Ernesto Vigna, Fortunato Morabito, Massimo Gentile

**Affiliations:** ^1^ Hematology Unit, Department of Onco‐Hematology AO of Cosenza Cosenza Italy; ^2^ Emergency and Internal Medicine Department Saint Joseph Hospital East Jerusalem Palestine; ^3^ Department of Experimental and Clinical Medicine University of Catanzaro Catanzaro Italy; ^4^ AIL Sezione di Cosenza Cosenza Italy; ^5^ Department of Pharmacy, Health and Nutritional Science University of Calabria Rende Italy

**Keywords:** chemo‐free strategy, follicular lymphoma, target therapy

## Abstract

Follicular lymphoma (FL), traditionally considered an indolent yet incurable malignancy, is experiencing a substantial evolution in its therapeutic landscape with the emergence of chemo‐free treatment strategies. These novel approaches challenge conventional chemotherapy‐based paradigms and offer promising alternatives for both newly diagnosed and relapsed/refractory (RR) FL patients. Among these innovations, bispecific antibodies (BsAbs) have demonstrated compelling efficacy while providing practical advantages, including outpatient administration and generally manageable safety profiles. Chimeric antigen receptor (CAR) T‐cell therapies have further expanded the therapeutic armamentarium, achieving unprecedented response rates in heavily pretreated and high‐risk populations, although their implementation remains limited by logistical complexity and high associated costs. Additional targeted agents—such as Enhancer of zeste homolog 2 (EZH2) inhibitors, lenalidomide, and Bruton tyrosine kinase (BTK) inhibitors—also contribute meaningfully to chemo‐free treatment options, particularly within combination regimens that may enhance clinical benefit. Despite these advances, several challenges persist. Early disease progression (POD24) remains one of the most powerful prognostic determinants in FL. The FLIPI‐C model, incorporating machine‐learning–derived risk stratification, has shown promise in identifying high‐risk patients who may benefit most from innovative approaches. Introducing chemo‐free therapies earlier in the treatment algorithm may improve outcomes for these patients while mitigating the long‐term toxicities associated with conventional chemotherapy. Ongoing validation through prospective clinical trials and real‐world evidence will be essential to define the optimal integration of these therapies. Overall, this evolving paradigm highlights the urgent need for continued innovation, multidisciplinary collaboration, and equitable access to ensure that the full potential of chemo‐free strategies can be realized for patients with this complex disease.

## 1 Introduction

Follicular lymphoma (FL) is the second most common subtype of non‐Hodgkin lymphoma, representing 20%–30% of all cases [1]. Despite progress in diagnostic techniques, FL typically presents at an advanced stage. Although the disease generally follows an indolent course and demonstrates high sensitivity to treatment, it remains incurable, and patients are at continual risk of relapse [2]. Traditional immunochemotherapy (ICT) regimens provide substantial disease control; however, their failure to significantly improve overall survival (OS) and their association with cumulative long‐term toxicities underscore the need for novel therapeutic strategies. Although most patients with follicular lymphoma respond to first‐line immunochemotherapy, approximately 20%–30% experience Progression of disease within 24 months (POD24), which is associated with poor long‐term survival.

In recent years, FL management has undergone a profound shift with the introduction of chemo‐free therapies. Bispecific antibodies (BsAbs) and chimeric antigen receptor T‐cell (CAR‐T) therapies [3] have emerged as highly effective options in relapsed/refractory FL (RR‐FL), offering alternatives for patients who are ineligible for chemotherapy. BsAbs, characterized by convenient administration schedules and manageable safety profiles, are expected to gain wider use, particularly in fixed‐duration regimens. CAR‐T therapies, albeit more resource‐intensive, provide the possibility of durable remissions and may ultimately challenge the long‐held perception of FL as an incurable malignancy.

The limitations of existing prognostic models further illustrate the complexities of FL management. The Follicular Lymphoma International Prognostic Index (FLIPI) [4], developed before the rituximab era and designed to predict OS, does not incorporate molecular biomarkers and is influenced by variability in radiological assessment. FLIPI‐2 [5], created to predict progression‐free survival (PFS) in patients treated with rituximab‐based therapies, similarly lacks integration of molecular features and therefore does not fully capture the biological heterogeneity of FL. Simplified clinical scores, including the PRIMA‐derived tool [6, 7], rely exclusively on clinical variables from cohorts treated with rituximab maintenance, further reinforcing the absence of molecular parameters [8].

Post hoc markers such as POD24, which identify high‐risk patients who progress within 24 months of frontline therapy [9, 10], are highly prognostic but cannot be assessed at baseline, restricting their applicability to newly diagnosed cases. More advanced models, including m7‐FLIPI and POD24‐PI, integrate mutational and clinical variables [11], improving prognostic precision but facing limitations related to cost, complexity, and limited availability outside specialized centers. These challenges have driven growing interest in prognostic frameworks that incorporate genetic alterations, microenvironmental characteristics, and treatment‐specific variables.

Traditional systems such as FLIPI, FLIPI‐2, and PRIMA Prognostic Index (PRIMA‐PI) remain inadequate for modern immunotherapy‐based strategies as they insufficiently reflect FL's molecular diversity. Emerging machine‐learning–based models, which incorporate recurrent mutations [e.g., Enhancer of zeste homolog 2 (EZH2), present in approximately 20%–25% of patients, A1.1 Histone‐lysine *N*‐methyltransferase 2D (KMT2D), TNF Receptor Superfamily Member 14 (TNFRSF14)] and immune‐interaction signatures, aim to identify patients who may benefit from early introduction of chemo‐free regimens such as BsAbs, lenalidomide‐based combinations, or tafasitamab‐containing approaches. Early evidence suggests that these biologically enriched models may outperform purely clinical indices in predicting early progression and guiding therapeutic decision‐making. However, standardized and broadly accessible tools for clinical application are still lacking [12].

The risk of histologic transformation of FL into aggressive lymphoma, most commonly diffuse large B‐cell lymphoma (DLBCL), represents another major clinical challenge. Transformation occurs at an annual rate of approximately 2%, with a poor 5‐year survival of 30%–50% [13]. Predicting which patients will transform remains difficult and significantly affects therapeutic strategy. Numerous investigations have explored potential biomarkers—genetic mutations, gene‐expression signatures, microRNAs, and microenvironmental factors—to predict transformation risk [14]. Despite extensive research, no single biomarker has demonstrated sufficient reliability for routine clinical use. A composite predictive model including Histone Cluster 1, H1e (HIST1H1E), KMT2D, and TNFRSF14 mutations, integrated with clinical variables, has shown promise in estimating individual transformation risk [15]; nonetheless, genetic heterogeneity and methodological inconsistencies across studies continue to impede clinical implementation.

For several decades, chemotherapy has constituted the backbone of FL treatment and remains the standard first‐line approach for patients with high tumor burden or symptomatic disease, particularly when combined with anti‐CD20 monoclonal antibodies [16, 17]. Landmark studies demonstrated that rituximab‐containing ICT significantly improves outcomes compared with chemotherapy alone [18, 19, 20, 21, 22, 23, 24]. ICT remains a common approach for RR‐FL, often employing non–cross‐resistant regimens or, in select cases, intensified strategies such as autologous stem cell transplantation (ASCT). However, response rates diminish with each successive line of therapy, and survival outcomes progressively decline. In a real‐world analysis, the median PFS after third‐line therapy was approximately 11 months, decreasing to around 4 months by the fifth line [25].

Notably, ICT has not yielded significant OS improvement across major clinical trials, including FOLL05 (comparing ICT regimens) [24], GALLIUM (comparing obinutuzumab versus rituximab) [26], and studies assessing rituximab maintenance versus observation [27]. These findings emphasize the pressing need for alternative, less toxic, and more durable treatment strategies.

The emergence of chemotherapy‐sparing strategies—including novel immune‐ and targeted‐therapy–based regimens—represents a paradigm shift in FL management. These agents aim to preserve or enhance therapeutic efficacy while reducing cumulative toxicities associated with chemotherapy.

Recently, accumulating evidence suggests that bispecific antibodies may transition from late‐line treatment to earlier phases of therapy. CD20 × CD3 BsAbs, initially approved for third‐line RR‐FL, are now being investigated in first‐line and second‐line settings in combination with agents such as lenalidomide, Bruton Tyrosine Kinase (BTK) inhibitors, and EZH2 inhibitors. These combinations aim to exploit synergistic immune activation and may offer deep, durable remissions without cumulative myelotoxicity. Notably, BsAbs appear to demonstrate comparable efficacy in POD24 and non‐POD24 patients, suggesting their potential value in biologically high‐risk subgroups traditionally less responsive to chemoimmunotherapy. The ultimate question of whether BsAbs can safely and effectively replace upfront chemotherapy will require mature long‐term data and may depend on the incorporation of response‐adapted strategies informed by circulating tumor DNA (ctDNA) [28].

**FIGURE 1:**
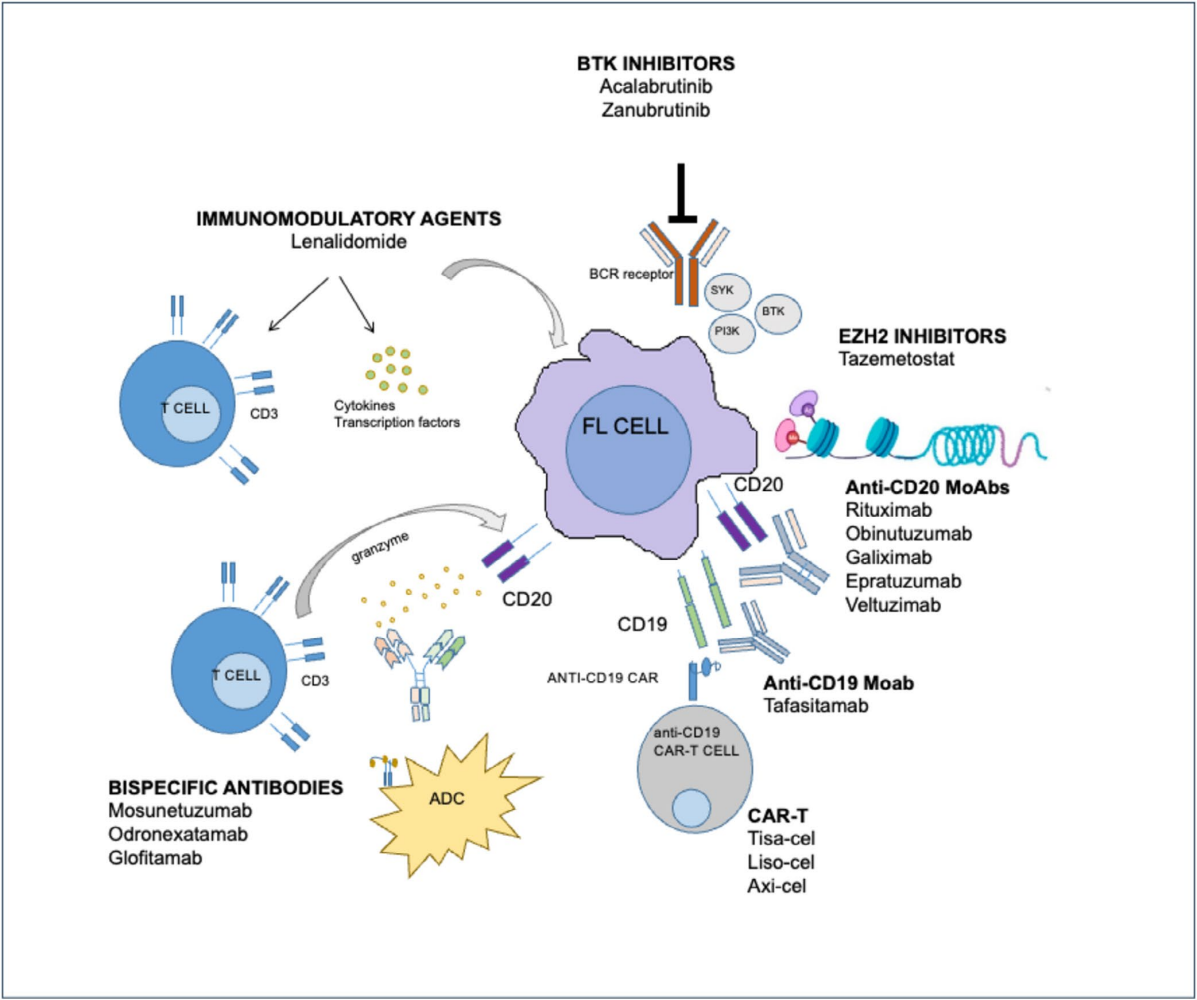
The figure summarizes the main chemo‐free strategies currently available, highlighting their distinct mechanisms of action and potential positioning across different disease phases. Anti‐CD20 monoclonal antibodies induce direct cytotoxicity and antibody‐dependent cellular cytotoxicity; immunomodulatory combinations enhance immune effector function within the tumor microenvironment; targeted agents inhibit key oncogenic or epigenetic pathways; bispecific antibodies redirect T cells toward malignant B cells through CD3–CD20 engagement; CAR T‐cell therapy mediates antigen‐specific cytotoxicity via engineered T‐cell receptors.

This review integrates current evidence on novel immune‐ and targeted‐therapy strategies that are redefining treatment paradigms in follicular lymphoma by minimizing reliance on cytotoxic chemotherapy and evaluates their efficacy, safety, and the logistical factors influencing their implementation in clinical practice. Through their novel mechanisms of action (Figure 1), these therapies may redefine current treatment algorithms and better meet unmet clinical needs, especially for patients with relapsed disease or those ineligible for chemotherapy.

## 2 Monoclonal Antibody Therapy

In patients with follicular lymphoma (FL) who are asymptomatic and have a low tumor burden, systemic treatment is traditionally deferred until the development of symptoms or organ compromise, without adversely affecting overall survival—a strategy known as “watchful waiting” [29, 30]. However, emerging evidence challenges this paradigm. Rituximab monotherapy, particularly when administered with maintenance therapy, can improve quality of life (QoL) with minimal toxicity, although without conferring a survival advantage [31].

In newly diagnosed, low–tumor burden FL patients who respond to rituximab induction, maintenance therapy may be unnecessary, as re‐treatment at progression provides comparable outcomes [32]. QoL is a critical consideration in FL [33], given the potential toxicities of treatment [34] and the lack of clear survival benefit with early intervention. Importantly, a recent update demonstrated that rituximab does not negatively affect QoL in patients with advanced‐stage, asymptomatic FL [35]. Consistent with earlier findings showing that rituximab monotherapy—whether induction alone or induction followed by maintenance—significantly prolongs PFS and reduces the need for subsequent treatments compared with watchful waiting [31], this updated analysis highlights improvements in selected QoL domains. Long‐term follow‐up (median 12.3 years) confirmed that rituximab effectively delays the need for additional therapy. Future research should focus on identifying, at baseline, those patients most likely to benefit from early rituximab to optimize QoL outcomes. Given its favorable safety profile (18 serious adverse events among 276 patients), treatment decisions should be individualized. In patients at low risk for histologic transformation and POD24, a patient‐centered discussion weighing observation versus rituximab remains essential.

In symptomatic patients, rituximab monotherapy demonstrates robust activity, with overall response rates (ORR) of 50%–70% and event‐free survival (EFS) ranging from 1 to 3 years [36, 37, 38, 39, 40, 41]. Its clinical efficacy was first established in a pivotal trial involving heavily pretreated individuals with relapsed or refractory indolent non‐Hodgkin lymphoma, yielding an ORR of 48% [38]. Additional evidence from the Swiss Group for Clinical Cancer Research (SAKK) [39, 40, 41] and the Nordic Lymphoma Group (NLG) [42, 43] suggests that many FL patients may safely avoid front‐line chemotherapy.

SAKK 35/98 compared standard rituximab induction with prolonged administration, showing that extended dosing significantly improved EFS—particularly in chemotherapy‐naïve patients—with long‐term follow‐up revealing a survival plateau among responders [40]. SAKK 35/03 evaluated maintenance rituximab for up to 5 years, demonstrating improved PFS but increased toxicity, raising concerns about routine long‐term maintenance [41].

A recent study comparing intravenous and subcutaneous rituximab in low‐burden FL demonstrated similar efficacy with improved convenience and healthcare resource utilization for the subcutaneous formulation. Combination strategies such as rituximab plus interferon‐α2a initially demonstrated enhanced response rates and prolonged survival, but toxicity limited their long‐term viability [42, 43, 44]. The success of rituximab has fuelled interest in next‐generation anti‐CD20 monoclonal antibodies, such as ofatumumab [45]. In a multicentre study of rituximab‐refractory FL, ofatumumab monotherapy (500 mg or 1000 mg) achieved an ORR of 11% and a median PFS of 5.8 months. Nearly half of patients achieved tumor reduction at 3 months, correlating with longer PFS (median 9.1 months). Ofatumumab was well tolerated, with infections, rash, and fatigue being the most frequent toxicities [46].

The HOMER study compared ofatumumab with rituximab in 438 patients previously treated with rituximab. Ofatumumab did not demonstrate superiority, with lower ORR (50% vs. 66%) and shorter median PFS (16.3 vs. 21.3 months), as well as higher rates of grade ≥ 3 adverse events [47]. These findings reflect the challenge of overcoming rituximab resistance, potentially linked to CD20 downregulation, despite ofatumumab's enhanced complement‐dependent cytotoxicity.

In previously untreated, low‐intermediate risk FL (CALGB 50901), ofatumumab 1000 mg yielded an ORR of 84% and a median PFS of 1.9 years, with good tolerability [48]. Despite the small sample size and design modifications, these results support its activity in front‐line settings.

More recently, the FIL‐MIRO study examined the role of ofatumumab as MRD‐guided consolidation after radiotherapy in early‐stage FL. Among MRD‐positive patients post‐radiotherapy, 92% achieved MRD negativity following ofatumumab, which correlated with reduced relapse risk and improved PFS [49].

Veltuzumab, a humanized anti‐CD20 antibody, has shown encouraging activity with both intravenous and subcutaneous administration [50]. Among 55 FL patients, median PFS was 6.2 months; responders had a median duration of response (DoR) of 10.2 months and PFS of 15.2 months, with some durable long‐term responses.

Tafasitamab (MOR208), an Fc‐engineered anti‐CD19 antibody, may be advantageous in tumors with reduced CD20 expression. In a Phase IIa study including 34 FL patients, tafasitamab achieved an ORR of 29% with responses lasting > 12 months in almost half of responding patients (4/9), and a median PFS of 8.8 months. Treatment was well tolerated, with infusion reactions and neutropenia as the most common adverse events [51, 52].

Polatuzumab vedotin (targeting CD79b) [53] and pinatuzumab vedotin (targeting CD22) [54] are antibody–drug conjugates (ADCs) delivering MMAE payloads. In the ROMULUS study, polatuzumab–rituximab (R‐pola) achieved an ORR of 70% and a CR rate of 45% in FL—higher than rituximab monotherapy historically—while pinatuzumab–rituximab (R‐pina) yielded an ORR of 62% but with a lower CR rate (5%). Median PFS was longer with R‐pola (15.3 vs. 12.7 months) [55].

## 3 Lenalidomide and Bruton's Tyrosine Kinase Inhibitors

Lenalidomide demonstrates efficacy as monotherapy and in combination with rituximab, providing a chemotherapy‐free alternative in FL [56]. As a single agent (25 mg/day), lenalidomide yielded an ORR of 23% and a median PFS of 4.4 months in heavily pretreated patients, highlighting the need for combination approaches [56].

The addition of rituximab (*R* ^2^ regimen) markedly improves outcomes. In rituximab‐refractory FL, *R* ^2^ produced an ORR of 65% (35% CR) with a median PFS of 16.5 months [57]. The Alliance trial confirmed *R* ^2^ superiority over lenalidomide monotherapy (ORR 76% vs. 53%) without a significant increase in toxicity [58].

The Phase III RELEVANCE trial demonstrated comparable long‐term outcomes between *R* ^2^ and R‐chemotherapy in untreated FL, with similar 6‐year PFS (60% vs. 59%) and OS (89% for both). R‐chemo showed slightly higher ORR and CR but with no survival benefit. *R* ^2^ avoids chemotherapy‐related toxicity and remains a valuable option for older and comorbid patients [59].

The prognostic role of POD24 has gained importance in the chemo‐free era. Analysis of SAKK trials confirmed POD24 as a strong predictor of outcome irrespective of treatment modality, underscoring its value for patient stratification in chemo‐free regimens [60].

The Phase III AUGMENT trial demonstrated clear superiority of *R* ^2^ over rituximab alone in RR FL, improving median PFS (39.4 vs. 14.1 months) and response rates (ORR 78% vs. 53%; CR 34% vs. 18%). Neutropenia was the most frequent grade ≥ 3 toxicity. AUGMENT established *R* ^2^ as standard therapy in RR FL [61, 62].

Real‐world data confirm these findings, with ORR 82% and median PFS 22 months. Importantly, patients excluded from AUGMENT—such as those with rituximab‐refractory disease—also benefited, supporting broad applicability. Bulky disease and rituximab refractoriness were independent negative prognostic factors [63].

The ongoing MAGNIFY study reports high response rates (ORR 72%; CR/complete response unconfirmed (CRu) 42%) and prolonged PFS (> 50 months) for *R* ^2^ induction in RR FL [64].

The inMIND trial added tafasitamab to *R* ^2^, significantly improving PFS (22.4 vs. 13.9 months) and response rates (ORR 83.5% vs. 72.4%; CR 49.4% vs. 39.8%), with a manageable safety profile [65]. The GALEN regimen (obinutuzumab‐lenalidomide) has demonstrated strong activity both in RR FL (2‐year PFS 65%; CR 38%) [66] and front‐line disease (ORR 94%; CR 80%; 3‐year PFS 82%) with predominantly haematologic toxicity [67]. A Phase Ib study of acalabrutinib plus rituximab and lenalidomide showed an ORR of 75.9%, with neutropenia as the most common severe adverse event, supporting further evaluation [68]. The ROSEWOOD trial demonstrated that zanubrutinib‐obinutuzumab significantly outperformed obinutuzumab alone, with ORR 69% vs. 46%, CR 39% vs. 19%, and median PFS 28.0 vs. 10.4 months. Toxicities were manageable, and serious events such as atrial fibrillation and major bleeding were rare [69].

## 4 Mutation‐Driven Therapy

Tazemetostat, an oral EZH2 inhibitor, is emerging as a key therapeutic option for relapsed or refractory follicular lymphoma (RR‐FL), offering clinically meaningful activity with a favorable safety profile. An initial phase 2 trial demonstrated its efficacy across molecular subgroups [70]. Among 99 treated patients, the ORR was 69% in those with EZH2‐mutated (EZH2^mut^) disease and 35% in those with wild‐type EZH2 (EZH2^WT^). Median progression‐free survival (PFS) was 13.8 months for EZH2^mut^ and 11.1 months for EZH2^WT^, with median durations of response (DOR) of 10.9 and 13.0 months, respectively.

A subsequent matched analysis further confirmed robust activity in both molecular cohorts, with ORRs of 71% (EZH2^mut^) and 50% (EZH2^WT^) and comparable PFS after adjustment for baseline differences (14.8 months vs. 14.3 months) [71]. Treatment‐related adverse events (AEs), including thrombocytopenia, neutropenia, and anemia, were manageable, and no treatment‐related deaths were reported. Collectively, these findings support tazemetostat as an important therapeutic option, particularly for patients unsuitable for cytotoxic or more intensive immune‐based approaches.

## 5 Bispecific Antibodies

Bispecific antibodies (BsAbs) represent a transformative therapeutic class in FL, where they have rapidly gained momentum in clinical development. Several agents—including mosunetuzumab, glofitamab, epcoritamab, and odronextamab—have demonstrated substantial efficacy in RR‐FL, both as monotherapy and in combination regimens [72, 73].

Mosunetuzumab, a first‐in‐class CD20 × CD3 bispecific antibody, is a major advance in immunotherapy for RR‐FL. A phase I study confirmed its feasibility and acceptable safety profile [74]. Step‐up dosing safely mitigated cytokine release syndrome (CRS). Among 197 patients treated in the step‐up cohort, neutropenia (28.4%), CRS (27.4%), and hypophosphatemia (23.4%) were common AEs. Grade ≥ 3 CRS was infrequent (2%), and no immune‐effector cell neurotoxicity was reported. In 65 evaluable FL patients, the ORR was 69.2%, with a complete response (CR) rate of 50.8%. Efficacy extended to high‐risk subgroups, including those previously treated with CAR T cells or refractory to both anti‐CD20 antibodies and PI3K inhibitors.

Mosunetuzumab received approval based on a phase 2 trial in 90 patients with a median of three prior therapies [75]. The ORR was 80%, with a CR rate of 60%, a median PFS of 17.9 months, and a median DOR of 22.8 months. CRS occurred in 44% (mostly grade 1–2), while grade 3–4 AEs were uncommon. Long‐term follow‐up at 37.4 months demonstrated sustained activity, with a median DOR of 35.9 months and estimated 36‐month overall survival (OS) of 82% [76]. Real‐world data support these findings, showing comparable PFS and slightly lower ORR and CR rates [77].

Epcoritamab, a subcutaneous CD3 × CD20 bispecific antibody, has shown deep and durable responses. In the EPCORE NHL‐1 study (median follow‐up 17.4 months), the ORR was 82%, with a CR rate of 62.5% [78, 79]. High rates of minimal residual disease (MRD) negativity correlated with prolonged PFS. CRS was predominantly low grade (grade 1–2: 65%; grade 3: 2%), and no grade ≥ 4 CRS occurred. Efficacy was consistent in high‐risk subgroups, including double‐refractory and early‐relapsing disease.

Odronextamab, another off‐the‐shelf CD20 × CD3 bispecific antibody, demonstrated strong activity in 115 patients previously treated with ≥ 2 lines of therapy [80]. The ORR was 80%, with a CR rate of 73%. Median DOR and PFS were 22.6 and 20.7 months, respectively. Safety was manageable, with mostly low‐grade CRS and infrequent discontinuations due to AEs.

Glofitamab, a CD20 × CD3 BsAb with a unique 2:1 CD20‐binding configuration, showed high efficacy in RR‐FL. Monotherapy produced an ORR of 81% and a complete metabolic response rate of 70%, while combination with obinutuzumab increased the ORR to 100% and the complete metabolic response to 74% [81]. However, combination therapy resulted in higher rates of myelosuppression and CRS.

Emerging data support shifting CD20 × CD3 BsAbs earlier in the therapeutic algorithm. Combinations with lenalidomide or EZH2 inhibitors may enhance response depth and durability—particularly in patients with preserved T‐cell function—permitting fixed‐duration, chemotherapy‐free regimens [82].

## 6 CD19‐Directed CAR T‐Cell Therapy

CAR T‐cell therapy, which employs genetically engineered autologous T cells expressing a CD19‐specific chimeric antigen receptor, has reshaped the treatment of RR‐FL [73, 83]. Three commercial CAR T products—axi‐cel, tisa‐cel, and liso‐cel—have demonstrated exceptional efficacy.

In the ZUMA‐5 trial, axi‐cel achieved an ORR of 94% and a CR rate of 79% in heavily pretreated RR‐FL [81, 82, 84]. CRS occurred in 78% (grade ≥ 3: 6%) and neurologic events in 56% (grade ≥ 3: 15%). Long‐term data showed a median DOR of 38.6 months and a median PFS of 40.2 months, with a 36‐month OS of 75% [85]. Real‐world outcomes from the CIBMTR Registry confirm similar efficacy and toxicity profiles [86].

In the ELARA trial, tisa‐cel produced an ORR of 86.2% and a CR rate of 69.1% [87]. At 12 months, estimated PFS was 67%. Updated results at 24 months reported PFS, DOR, and OS rates of 57.4%, 66.4%, and 87.7%, respectively [88]. Biomarker analyses suggest that reduced T‐cell exhaustion and higher naïve CD8+ T‐cell prevalence predict better responses.

The TRANSCEND FL study, the largest CAR T trial in FL, demonstrated an ORR of 97% and a CR rate of 94% with liso‐cel [85]. Severe CRS (1%) and neurotoxicity (2%) were rare, and some patients were treated in the outpatient setting. Median PFS and DOR were unreached at 17 months of follow‐up.

Real‐world data from CIBMTR and DESCAR‐T registries reinforce the efficacy and safety of axi‐cel and tisa‐cel in broader patient populations [86]. Ongoing randomized trials (ZUMA‐22, LEDA) will determine the role of CAR T therapy versus standard approaches in earlier lines [89, 90].

## 7 Circulating Tumor DNA Monitoring in Follicular Lymphoma

ctDNA monitoring is emerging as a powerful and minimally invasive approach for dynamic risk stratification and response assessment in follicular lymphoma (FL). Baseline ctDNA levels correlate with tumor burden and adverse clinical features, and high pretreatment ctDNA has been associated with an increased risk of early progression, including POD24. Importantly, longitudinal ctDNA assessment during therapy provides real‐time insight into treatment efficacy, with early ctDNA clearance strongly predicting durable remissions and improved progression‐free survival [25, 91].

Highly sensitive next‐generation sequencing–based techniques, such as phased variant enrichment and immunoglobulin gene sequencing, allow detection of minimal residual disease at levels far below the sensitivity of conventional imaging or PCR‐based BCL2–IGH assays. In the context of emerging chemo‐free strategies, ctDNA monitoring may enable response‐adapted approaches, identifying patients suitable for treatment de‐escalation or fixed‐duration therapy, while persistent or rising ctDNA could support early therapeutic intensification with bispecific antibodies or CAR T‐cell therapy. Ongoing prospective studies incorporating ctDNA‐guided endpoints are expected to clarify its role in personalized treatment algorithms for FL [92].

## 8 Conclusions

**FIGURE 2:**
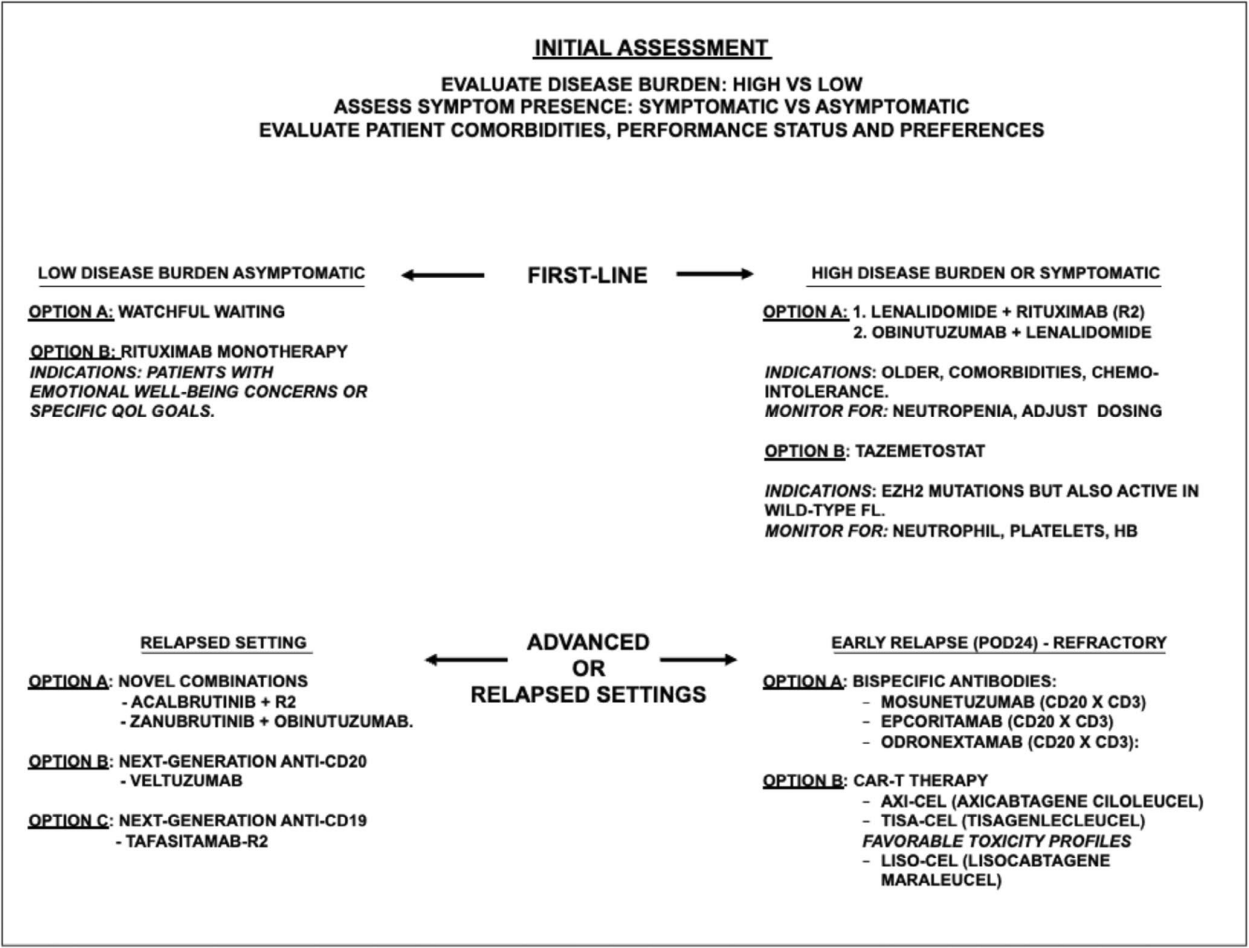
Proposed chemo‐free therapeutic algorithm for follicular lymphoma management.

In the current era of expanding chemo‐free options for follicular lymphoma, the proposed therapeutic algorithm integrates clinical, biological, and treatment‐related variables to guide personalized decision‐making across different disease phases. Key clinical factors include tumor burden, symptomatic status, patient age, comorbidities, and fitness for intensive or cellular therapies. Disease‐related variables such as POD24 status, risk of histologic transformation, and prior treatment exposure play a central role in identifying high‐risk patients who may benefit from early therapeutic intensification. Molecular and dynamic biomarkers, including EZH2 mutational status and emerging tools such as ctDNA monitoring, may further refine risk stratification and support response‐adapted strategies. Finally, treatment‐specific considerations—such as expected depth and durability of response, toxicity profiles, logistical complexity, and access to specialized centers—are critical when selecting among bispecific antibodies, lenalidomide‐based combinations, targeted agents, and CAR T‐cell therapy. Collectively, these variables inform a flexible, risk‐adapted algorithm aimed at maximizing efficacy while minimizing cumulative toxicity and overtreatment (Figure 2). The therapeutic landscape of FL is undergoing a paradigm shift, driven by the rapid emergence of highly effective, chemotherapy‐free treatments. These modalities—spanning bispecific antibodies, CAR T‐cell therapy, lenalidomide‐based combinations, and molecularly targeted agents—are reshaping expectations for disease control and long‐term survivorship. Figure 2 illustrates a proposed chemo‐free approach integrating established and investigational non‐chemotherapeutic strategies across first‐line and relapsed settings, tailored according to disease burden and patient risk.

Validation of a chemo‐free approach will require rigorously designed clinical trials and robust real‐world evidence. Integrating these therapies earlier may improve outcomes for high‐risk patients while reducing exposure to cytotoxic agents. Nonetheless, the prediction of POD24 remains challenging. Recently, the machine‐learning–based FLIPI‐C model demonstrated superior performance in predicting POD24 compared with traditional prognostic tools, with AUCs of 0.764 (training) and 0.703 (validation, GALLIUM) [90].

Simultaneously, ctDNA‐based monitoring is emerging as a powerful tool for dynamic risk stratification and response assessment. Highly sensitive sequencing modalities—such as phased variant enrichment—can detect minimal residual disease at far lower thresholds than traditional BCL2–IGH PCR assays. Pretreatment ctDNA burden correlates strongly with risk of early progression and may outperform existing clinical scores. In chemo‐free regimens, ctDNA clearance may identify patients eligible for de‐escalation or shorter fixed‐duration therapy, whereas persistent ctDNA could guide early intensification with CAR T cells or BsAb‐based combinations. Ongoing trials integrating ctDNA endpoints are expected to redefine risk‐adapted strategies in FL [82].

From an expert standpoint, the field is entering a decisive moment: the convergence of potent immune‐based therapies, molecular risk stratification, and real‐time genomic monitoring offers an unprecedented opportunity to personalize FL management beyond traditional algorithms (Table 1). To fully realize these benefits, sustained innovation, multidisciplinary collaboration, and equitable access to advanced treatments will be essential. Continued integration of biologically informed strategies is likely to move the field closer to the long‐term goal of durable remission—potentially for selected patients, without the need for chemotherapy.

**TABLE 1:** Clinical studies in follicular lymphoma.

Therapeutic class	Regimen	Study	Patient population	Line of therapy	Main outcomes
Anti‐CD20 monoclonal antibody	Rituximab (monotherapy)	Ardeshna et al., Phase III	Low‐tumor burden, asymptomatic FL	First‐line	Improved PFS and QoL vs. watchful waiting; no OS benefit
Immunomodulatory therapy	Lenalidomide + rituximab (*R* ^2^)	RELEVANCE, Phase III	Untreated FL	First‐line	Comparable PFS and OS to R‐chemotherapy with reduced chemotherapy‐related toxicity
Immunomodulatory therapy	Lenalidomide + rituximab (*R* ^2^)	AUGMENT, Phase III	RR‐FL	Relapsed/Refractory	Superior PFS and response rates vs. rituximab alone
Targeted therapy (EZH2 inhibitor)	Tazemetostat	Phase II	RR‐FL (EZH2‐mut and EZH2‐WT)	≥ 2 lines	Higher ORR in EZH2‐mut FL; favorable safety profile
Bispecific antibody (CD20 × CD3)	Mosunetuzumab	Phase II	RR‐FL	≥ 3 lines	High CR rates with manageable CRS; durable responses
Bispecific antibody (CD20 × CD3)	Epcoritamab	EPCORE NHL‐1, Phase I/II	RR‐FL	≥ 2 lines	Deep and durable responses; high MRD negativity
Bispecific antibody (CD20 × CD3)	Glofitamab	Phase I/II	RR‐FL	≥ 2 lines	High response rates; increased CRS with combination strategies
CAR T‐cell therapy (CD19)	Axicabtagene ciloleucel (axi‐cel)	ZUMA‐5, Phase II	RR‐FL	≥ 2 lines	Very high ORR and CR; durable remissions
CAR T‐cell therapy (CD19)	Tisagenlecleucel (tisa‐cel)	ELARA, Phase II	RR‐FL	≥ 2 lines	High efficacy with low rates of severe CRS and neurotoxicity
CAR T‐cell therapy (CD19)	Lisocabtagene maraleucel (liso‐cel)	Lisocabtagene maraleucel (Phase II)	RR‐FL	≥ 2 lines	High CR rates with a favorable safety profile

Abbreviations: CR, complete response; CRS, cytokine release syndrome; DoR, duration of response; FL, follicular lymphoma; MRD, minimal residual disease; ORR, overall response rate; OS, overall survival; PFS, progression‐free survival; QoL, quality of life; RR‐FL, relapsed/refractory follicular lymphoma.

## Author Contributions

**Enrica Antonia Martino**, **Santino Caserta**, **Mamdouh Skafi**, **Fortunato Morabito**, **Massimo Gentile:** conceptualization. **Enrica Antonia Martino**, **Francesco Mendicino**, **Ernesto Vigna**, **Antonella Bruzzese**, and **Fortunato Morabito:** methodology. **Enrica Antonia Martino**, **Santino Caserta**, **Fortunato Morabito**, **Massimo Gentile:** writing – original draft preparation. **Enrica Antonia Martino**, **Santino Caserta**, **Mamdouh Skafi**, **Fortunato Morabito**, **Massimo Gentile:** writing, review, and editing. All authors have read and agreed to the published version of the manuscript.

## Funding

The authors have nothing to report.

## Ethics Statement

The authors have nothing to report.

## Conflicts of Interest

The authors declare no conflicts of interest.

## Data Availability

Data sharing not applicable to this article as no datasets were generated or analysed during the current study.
